# Infection Control for a Carbapenem-Resistant Enterobacteriaceae Outbreak in an Advanced Emergency Medical Services Center

**DOI:** 10.3390/antibiotics10121537

**Published:** 2021-12-15

**Authors:** Yoshiro Sakai, Kenji Gotoh, Ryuichi Nakano, Jun Iwahashi, Miho Miura, Rie Horita, Naoki Miyamoto, Hisakazu Yano, Mikinori Kannae, Osamu Takasu, Hiroshi Watanabe

**Affiliations:** 1 Department of Pharmacy, Kurume University Hospital, Kurume 831-0011, Japan; sakai_yoshirou@kurume-u.ac.jp; 2 Department of Infection Control and Prevention, Kurume University School of Medicine, Kurume 831-0011, Japan; iwahashi@kurume-u.ac.jp (J.I.); hwata@med.kurume-u.ac.jp (H.W.); 3 Division of Infection Control and Prevention, Kurume University Hospital, Kurume 831-0011, Japan; miura_miho@kurume-u.ac.jp; 4 Department of Microbiology and Infectious Diseases, Nara Medical University, Nara 634-8521, Japan; rnakano@naramed-u.ac.jp (R.N.); yanohisa@naramed-u.ac.jp (H.Y.); 5 Department of Clinical Laboratory Medicine, Kurume University Hospital, Kurume 831-0011, Japan; horita_rie@kurume-u.ac.jp (R.H.); miyamoto_naoki@kurume-u.ac.jp (N.M.); 6 Department of Emergency and Critical Care Medicine, Kurume University School of Medicine, Kurume 831-0011, Japan; kannae_mikinori@med.kurume-u.ac.jp (M.K.); takasu_osamu@kurume-u.ac.jp (O.T.)

**Keywords:** carbapenem-resistant Enterobacteriaceae (CRE), outbreak, infection control, pulsed-field gel electrophoresis (PFGE), multilocus sequence typing (MLST), carbapenemase

## Abstract

Background: A carbapenem-resistant Enterobacteriaceae (CRE) outbreak occurred in an advanced emergency medical service center [hereafter referred to as the intensive care unit (ICU)] between 2016 and 2017. Aim: Our objective was to evaluate the infection control measures for CRE outbreaks. Methods: CRE strains were detected in 16 inpatients located at multiple sites. Environmental cultures were performed and CRE strains were detected in 3 of 38 sites tested. Pulsed-field gel electrophoresis (PFGE), multilocus sequence typing (MLST), and detection of β-lactamase genes were performed against 25 CRE strains. Findings: Molecular typing showed the PFGE patterns of two of four *Klebsiella pneumoniae* strains were closely related and the same MLST (ST2388), and four of five *Enterobacter cloacae* strains were closely related and same MLST (ST252). Twenty-three of 25 CRE strains harbored the IMP-1 β-lactamase gene and 15 of 23 CRE strains possessed IncFIIA replicon regions. Despite interventions by the infection control team, new inpatients with the CRE strain continued to appear. Therefore, the ICU was partially closed and the inpatients with CRE were isolated, and the ICU staff was divided into two groups between inpatients with CRE and non-CRE strains to avoid cross-contamination. Although the occurrence of new cases dissipated quickly after the partial closure, a few months were required to eradicate the CRE outbreak. Conclusion: Our data suggest that the various and combined measures that were used for infection control were essential in stopping this CRE outbreak. In particular, partial closure to isolate the ICU and division of the ICU staff were effective.

## 1. Introduction

Carbapenem-resistant Enterobacteriaceae (CRE) is a major health concern worldwide, including infections found in Japan [1,2,3]. Outbreaks of CRE have occurred in most parts of the world during the past decade [4,5,6]. When CRE is detected in hospitalized patients, contact precautions for infection control of CRE is very important. Hospitalized patients may be particularly susceptible to infections, and CRE infections are associated with increasing the risks of morbidity and mortality, prolonged hospital stay, and increasing health care costs [7,8]. As mentioned earlier, clinical infections with CRE are associated with high rates of morbidity and mortality, which is due, in part, to limited options for therapy [7]. When CRE is detected in hospitalized patients, contact precautions for infection control of CRE are very important [8]. The treatment options for CRE infections remain very limited, and colistin and tigecycline are considered the drugs of choice to treat infections caused by CRE [9]. However, the emergence of bacteria that are resistant to these antibiotics has also been recognized worldwide [10,11,12]. In addition, outbreaks of colistin-resistant CRE have occurred [11,13].

The management of CRE in hospital settings is not only costly but presents a significant challenge. While reliably detecting CRE in the laboratory is an important first step, it can be hampered by the fact that resistance occurs through a variety of different mechanisms. By accurately understanding the homology and resistance mechanisms of CRE, it is possible to know whether the infection is nosocomial or spreading in the community. This information is useful in deciding whether infection control measures should be implemented on a ward basis, on a hospital basis, or including the community [14]. On the other hand, knowing the mechanism of resistance can contribute to the appropriate selection of therapeutic agents. KPC-producing CRE can be treated with antimicrobial agents containing avibactam and vaborbactam, so colistin and tigecycline can be preserved [15,16]. This is also important from the viewpoint of antimicrobial stewardship.

The advanced emergency medical service center in Kurume University Hospital has experienced several outbreaks due to resistant bacteria such as methicillin-resistant *Staphylococcus aureus* and vancomycin-intermediate *S. aureus*, and an infection control team (ICT) is usually implemented [17,18]. In this report, we describe a CRE outbreak in our advanced emergency medical service center (hereafter referred to as the ICU) and discuss the stepwise infection control measures that were implemented, along with our evaluation of the effectiveness of these measures.

## 2. Results

### 2.1. Bacterial Strains and Patient Characteristics

**Figure 1:**
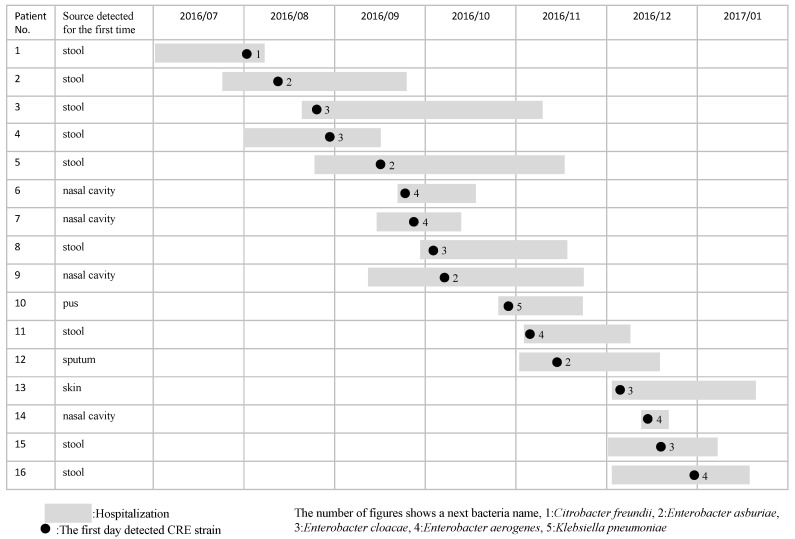
The time course for a CRE outbreak in the ICU. Gray shadow: period of hospitalization. Black circle: the first day of CRE strain detection.

Sixteen CRE strains were isolated from the stools of nine inpatients; from the nasal cavity of four inpatients; and from the pus, sputum, and skin of each of these inpatients between August and December 2016 (Figure 1). The mean age of the 16 inpatients (11 males and 5 females) was 65.7 years, and their actual ages ranged from 24 to 86 years. The mean detection period of the CRE strain after admission was 23.9 days, which represented a range of from 1 to 170 days. CRE had been detected at the point of hospitalization in 4 of the 16 inpatients. During the CRE outbreak, 13 CRE strains were recognized as colonization. However, three CRE strains were isolated from inpatients with pneumonia or bacteremia, and one inpatient died from bacteremia due to CRE.

An environmental culture was performed in November 2016, and CRE strains were detected in 3 of 38 sites (3 different sinks).

### 2.2. MIC

In judging the effectiveness of CRE treatments, eight isolates showed meropenem MICs ≥ 2 mg/L, another eight isolates showed imipenem MICs ≥ 2 mg/L, and cefmetazole recorded MICs ≥ 64 mg/L according to the reporting criteria of the Infectious Disease Act of Japan (Table 1).

### 2.3. Interpretation of Molecular Typing by PFGE and MLST Analysis

**Figure 2:**
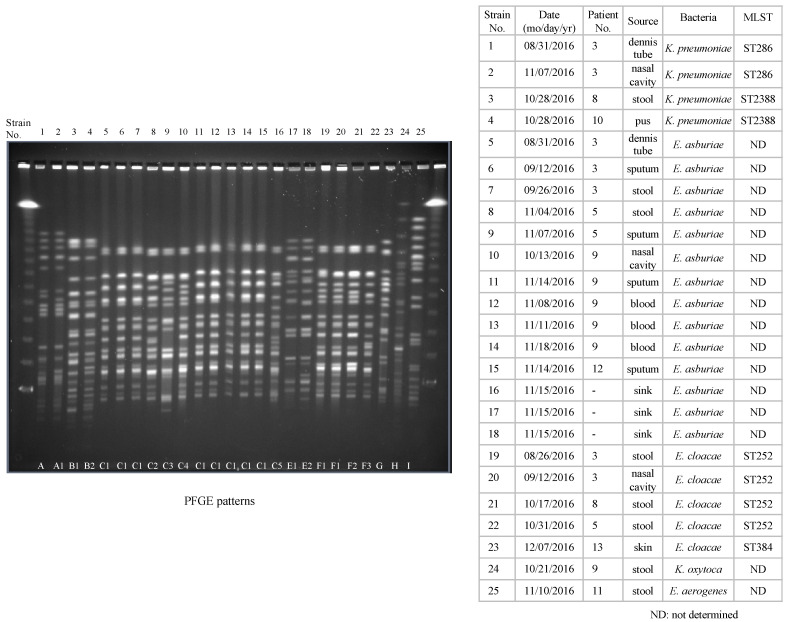
PFGE patterns of *Xba*I-digested DNA from 25 CRE isolates (22 from inpatients and 3 from environments). Molecular typing showed that the PFGE patterns of 25 CRE isolates were divided into eight patterns (A–I). Those of *K. pneumoniae* in strains three and four, *E. asburiae* from strains 5 to 16, and *E. cloacae* strains from strains No.19 to 22 were closely related. Similarly, the MLST patterns of 2 *K. pneumoniae* in strains three and four were identical as ST2388 and those of four *E. cloacae* strains from strains 19 to 22 were identical as ST252.

Molecular typing by the PFGE patterns of 25 CRE strains was divided into eight patterns (A–I). The PFGE patterns of *K. pneumoniae* in strains three and four, *Enterobacter asburiae* from strains 5 to 16, and *E. cloacae* from strains 19 to 22 all were closely related (Figure 2). MLST analysis was performed for all four of the *K. pneumoniae* strains and for 5 of the *E. cloacae* strains. Of *K. pneumoniae* the isolates identified ST286 (2 strains) and ST2388 (2 strains). Of *E. cloacae*, the isolates identified ST252 (4 strains) and ST384 (1 strain).

### 2.4. Distribution of β-Lactamase Genes

The distribution of β-lactamase genes is shown in Table 2. Twenty-three isolates were positive for CIM, and all of the isolates harbored the IMP-1 β-lactamase gene. Two other CIM-negative isolates harbored no carbapenemase gene and were categorized as non-CPE (carbapenemase-producing Enterobacteriaceae). These two strains were resistant to carbapenems probably due to overexpression of AmpC β-lactamase combined with a disrupted outer membrane (porin) permeability or other mechanisms.

Incompatibility group typing revealed two types of plasmids in the CPEs. Fifteen of the 23 CPE isolates possessed IncFIIA replicon regions, including *K. pneumoniae* (*n* = 2), *E. asburiae* (*n* = 11), and *E. cloacae* (*n* = 2). Two other isolates belonged to IncN in *K. pneumoniae*, and the remaining six isolates were not determined according to Inc type. We assumed that the IMP-1 β-lactamase gene encoding the IncFIIA plasmid was disseminated among the species.

### 2.5. Intervention by the ICT

Initiatives for cohort isolation, active surveillance, environmental culture, monitoring, and education for the ICU staff were performed by the ICT. However, new inpatients with the CRE strain continued to appear despite such interventions. We notified the government of the outbreak and received guidance, but the outbreak was not contained. We invited several additional infection control experts from other facilities to take measures, but the outbreak was still not contained. Therefore, the ICU was partially closed after discussions with the government in November 2016, and the inpatients with the CRE strain were isolated in order to prevent further horizontal transmission. The ICU staff was divided into two groups between inpatients with CRE and non-CRE strains to avoid cross-contamination. Although the occurrence of new cases dissipated quickly after the partial closure, it took several months to eradicate the CRE outbreak, and the hospital suffered economically.

## 3. Discussion

In this study, we characterized the epidemiological, microbiological, and molecular analysis of CRE outbreaks in an advanced emergency medical service center in Japan. CRE has recently been detected in the world and its outbreaks have increased [19]. Because CRE had been detected in 4 of the 16 inpatients before hospitalization, we have routinely performed surveillance cultures for all patients upon admission. Thus, it is important to monitor patients with resistant bacteria before hospitalization [8,20,21]. If there is an increasing trend in the frequency of isolates of resistant organisms such as CRE, vancomycin resistance *Enterococcus faecium* and vancomycin resistant *Staphylococcus aureus* in the tests at the time of admission, it is also necessary to exchange information and collaborate on the isolation status of resistant organisms at medical facilities in the surrounding areas.

The routes of infection for resistant organisms such as CRE are mainly the result of direct or indirect contact that can be spread in a ward via the transiently colonized hands of healthcare workers [8,20], and CRE is known to exist in hospital water environments such as sinks [6]. In addition, our results showed that the CRE organisms detected in blood culture and in the sink were identified as the same bacteria by MLST analysis. Furthermore, closely related strains have been detected in several different sinks. Considering the results, daily cleaning of hospital water environments [19,20,21] and hand hygiene [21,22] are important for infection control. In response to this outbreak, we took these factors into consideration and conducted daily rounds and infection control, focusing on cleaning the water environment. However, it was not enough to contain the CRE outbreak. The water-free ICU is now being proposed as a management method for the water-borne outbreak, and Some reports have shown that removing sinks in intensive care units has reduced the prevalence of multidrug-resistant Gram-negative bacteria [23,24]. Gram-negative bacteria can survive for a long time in sinks, where they acquire resistant genes through contact with bacteria that have resistant genes. By washing hands in the contaminated sink, water droplets are dispersed into the environment and adhere to the clothes of healthcare workers, which is thought to spread the drug-resistant bacteria in the ICU. If we had stopped using sinks, the CRE outbreak might have been contained earlier.

During the CRE outbreak, molecular analysis by PFGE was performed repeatedly to evaluate horizontal transmission, and members of the ICU staff were immediately informed of the results. PFGE seems useful for evaluating the presence of horizontal transmission in hospital-acquired infection [17,18]. Furthermore, we used MLST for analysis in addition to PFGE for a portion of the CRE strains. The two methods detected the same sequence in most strains.

In this study, 23 of 25 CRE strains produced IMP-1, but the remaining two isolates had no carbapenemase. Despite the fact that KPC, OXA-48, and NDM are found globally, these are rarely found in Japan, where IMP-1 and IMP-6 are exclusively the predominant forms of carbapenemases [25,26]. Since the resistance gene is known to spread across strains producing carbapenemase [27], infection control against inpatients with the CRE strain is important in preventing outbreaks. Our results also suggest that 17/23 strains of CPE had transmissible plasmid. Further, because the IMP-1 β-lactamase gene encoding the IncFIIA plasmid was disseminated among the species, infection control against CPE is particularly important regardless of the bacterial species. Regarding the choice of therapeutic agents, it is important to investigate the type of carbapenemase in CRE outbreaks. These CREs that produced IMP type carbapenemase cannot be treated with antibacterial agents including beta-lactamase inhibitors such as avibactam and vaborbactam. Because The CRE strains in this study were susceptible to aztreonam, quinolones, tetracycline, and aminoglycosides, we were able to intervene appropriately regarding the choice of treatment. The appropriate use of antimicrobial agents is essential to inhibit the emergence of resistant strains, including CRE.

During the CRE outbreak, active surveillance, environmental culture, monitoring, and education for the ICU staff were performed by the ICT, but we were unable to stop the expansion of CRE. As a result, we partially closed the ICU, which allowed us to strictly segregate staff caring for CRE-affected and unaffected inpatients. Nevertheless, several months were required to finally terminate the CRE outbreak, and the hospital suffered economically as a result.

This study is limited by the fact that it is a single-center experience of a CRE outbreak, and the number of cases is small. In order to prevent the spread of CRE after even one case is isolated, infection control measures and laboratory testing systems similar to those for outbreaks are necessary. Our analysis of the organisms and estimation of the route of infection will be useful for other institutions.

In conclusion, despite the employment of various infection control measures, partial closure for isolation plus division of the ICU staff was essential in terminating this CRE outbreak.

## 4. Methods

### 4.1. Ethical Approval

All studies described herein were approved by the Human Ethics Review Boards of Kurume University (17161). At the time of admission to the ICU, we have obtained consent from the patient or family for checking resistant organisms’ carriage and for active surveillance in all cases.

### 4.2. Setting and Outbreak Description

In the Kurume University Hospital, there are 25 diagnosis and treatment departments that serve 24 wards with 1018 beds, which includes an ICU with 43 beds. The ICU accepts many severe patients from ambulance and helicopter emergency medical services. A CRE strain was first detected from the stool of an inpatient in the ICU in August 2016. Isolation in a private room and the reinforcement of direct or indirect contact infection measures were performed for this inpatient with the CRE strain. However, additional inpatients with the CRE strain eventually emerged. Three new inpatients with the CRE strain were simultaneously identified at the beginning of September 2016, and the infection control team (ICT) classified the intervention with the status of an outbreak.

### 4.3. Bacterial Strains and Patients

Twenty-five CRE isolates from 16 inpatients and three environments in the ICU between August and December 2016 were enrolled in this study.

### 4.4. Identification Test and Minimum Inhibitory Concentration

An identification test was conducted using MicroScan WalkAway96 plus NBP 6.23J (Siemens Healthcare Diagnostics Inc., Tokyo, Japan). The minimum inhibitory concentrations (MIC) of ampicillin, piperacillin, cefotaxime, ceftazidime, cefepime, cefmetazole, imipenem, meropenem, aztreonam, ampicillin/sulbactam, piperacillin/tazobactam, gentamicin, amikacin, minocycline, levofloxacin, and sulfamethoxazole-trimethoprim were determined in reference to MicroScan Neg NENC1J (Siemens Healthcare Diagnostics Inc., Tokyo, Japan) via the broth-dilution method, in accordance with the guidelines of the Clinical and Laboratory Standards Institute [28]. The criteria for CRE were based on laboratory findings of Japanese criteria as follows: the MIC for meropenem was ≥2 mg/L, or the MIC for imipenem was ≥2 mg/L and the MIC for cefmetazole was ≥64 mg/L.

### 4.5. Pulsed-Field Gel Electrophoresis

Pulsed-field gel electrophoresis (PFGE) against 25 CRE strains (22 from inpatients and three from environments) was performed, as described previously [17]. The DNA was digested with *Xba*I (Takara Shuzo Co., Shiga, Japan). CHEF Mapper pulsed-field electrophoresis systems (Bio-Rad Life Science Group, Hercules, CA, USA) were used with a potential of 6 V/cm, with switch times of 2.16 and 44.69 s, and run-times of 20 h. After staining with ethidium bromide, the PFGE patterns were interpreted based on the criteria described by Tenover et al. [29,30].

### 4.6. Multilocus Sequence Typing

Multilocus sequence typing (MLST) was performed for the isolates of *Klebsiella pneumoniae* and *Enterobacter cloacae*. All strains of *K. pneumoniae* and *E. cloacae* were assessed by MLST in accordance with the protocol on the MLST website. The primers of seven housekeeping genes were based on information from the following website: https://pubmlst.org/ecloacae/, https://bigsdb.pasteur.fr/klebsiella/klebsiella.html (accessed on 10 July 2019). The sequence types were assigned using the MLST website.

### 4.7. Detection of β-Lactamase Genes

Carbapenemase production was confirmed using the carbapenem inactivation method (CIM) [31]. The presence of β-lactamase genes including carbapenemases (*bla*_IMP_, *bla*_VIM_, *bla*_KPC_, *bla*_OXA-48-like_, and *bla*_NDM_) and ESBL (*bla*_TEM_, *bla*_SHV_, and *bla*_CTX-M_) was assessed using PCR and DNA sequencing as previously described [32,33].

### 4.8. Plasmid Incompatibility Typing

Plasmids incompatibility (Inc) groups were determined using the PCR replicon-typing scheme, as previously described [34].

## 5. Conclusions

Despite the employment of various infection control measures, partial closure for isolation plus division of the ICU staff was essential in terminating this CRE outbreak.

## Figures and Tables

**Table 1:** Antibiotics susceptibolity profiles (minimum inhibitory concentrations, mg/L).

Patient No.	ABPC	PIPC	CTX	CAZ	CFPM	CMZ	IPM	MEPM	AZT	ABPC/SBT	PIPC/TAZ	GM	AMK	MINO	LVFX	ST
1	>16	<8	>2	>8	4	>32	2	>2	<4	>16	<16	4	<4	4	2	<2
2	>16	>64	>2	>8	>16	>32	>2	>2	<4	>16	64	>8	<4	4	1	<2
3	>16	>64	>2	>8	>16	>32	>2	>2	<4	>16	>64	>8	<4	4	1	<2
4	>16	<8	<1	<4	<2	>32	2	<1	<4	16	<16	<2	<4	4	<0.5	<2
5	>16	>64	>2	>8	>16	>32	>2	>2	>8	>16	>64	8	<4	<2	<0.5	<2
6	<8	<8	<1	<4	<2	>32	2	<1	<4	<8	<16	<2	<4	<2	<0.5	<2
7	>16	<8	<1	<4	<2	>32	2	<1	<4	16	<16	<2	<4	<2	<0.5	<2
8	>16	>64	>2	>8	>16	>32	>2	>2	>8	>16	>64	>8	<4	>8	4	<2
9	>16	>64	>2	>8	>16	>32	>2	>2	<4	>16	64	>8	<4	>8	4	>2
10	>16	16	>2	>8	>16	>32	2	>2	<4	>16	<16	8	<4	4	4	>2
11	>16	<8	<1	<4	<2	>32	2	<1	<4	16	<16	<2	<4	<2	<0.5	<2
12	>16	<8	>2	>8	4	>32	>2	>2	<4	>16	<16	>8	<4	<2	1	<2
13	>16	<8	2	<4	<2	>32	2	<1	<4	>16	<16	<2	<4	4	<0.5	<2
14	>16	<8	<1	<4	<2	>32	2	<1	<4	16	<16	<2	<4	<2	<0.5	<2
15	>16	<8	>2	<4	<2	>32	2	<1	<4	>16	<16	<2	<4	<2	<0.5	<2
16	>16	<8	<1	<4	<2	>32	2	<1	<4	16	<16	<2	<4	<2	<0.5	<2

ABPC: ampicillin, PIPC: piperacillin, CTX: cefotaxime, CAZ: ceftazidime, CFPM: cefepime, CMZ: cefmetazole, IPM: imipenem, MEPM: meropenem, AZT: aztreonam, ABPC/SBT: ampicillin/sulbactam, PIPC/TAZ: piperacillin/tazobactam, GM: gentamicin, AMK: amikacin, MINO: minocycline, LVFX: levofloxacin, ST: sulfamethoxazole-trimethoprim.

**Table 2:** Distribution of β-lactamase genes for 25 CRE isolates (22 from inpatients and 3 from environments). Twenty-three isolates were positive for CIM and all of the isolates harbored the IMP-1 β-lactamase gene. Two other CIM-negative isolates harbored no carbapenemase gene. Incompatibility group typing revealed two types of plasmids in the CPEs. Fifteen of the 23 CPE isolates possessed IncFIIA replicon regions, including *K. pneumoniae* (*n* = 2), *E. asburiae* (*n* = 11), and *E. cloacae* (*n* = 2). Two other isolates belonged to IncN in *K. pneumoniae*, and the Inc type could not be determined for the six remaining isolates.

Strain No.	Carbapenemase	CTX-M	ESBL	CIM	Inc
1	IMP-1	ND	SHV	+	FIIA
2	IMP-1	ND	SHV	+	FIIA
3	IMP-1	ND	SHV	+	N
4	IMP-1	ND	SHV	+	N
5	IMP-1	ND	ND	+	FIIA
6	IMP-1	ND	TEM, SHV	+	FIIA
7	IMP-1	ND	TEM, SHV	+	FIIA
8	IMP-1	ND	ND	+	FIIA
9	IMP-1	ND	SHV	+	ND
10	IMP-1	ND	TEM, SHV	+	FIIA
11	IMP-1	ND	ND	+	FIIA
12	IMP-1	ND	ND	+	FIIA
13	IMP-1	ND	ND	+	FIIA
14	IMP-1	ND	ND	+	FIIA
15	IMP-1	ND	ND	+	FIIA
16	IMP-1	ND	ND	+	FIIA
17	IMP-1	ND	ND	+	ND
18	IMP-1	ND	ND	+	ND
19	IMP-1	ND	TEM, SHV	+	ND
20	IMP-1	ND	TEM	+	ND
21	IMP-1	ND	TEM, SHV	+	FIIA
22	IMP-1	ND	TEM, SHV	+	FIIA
23	ND	ND	ND	-	ND
24	IMP-1	ND	TEM	+	ND
25	ND	ND	ND	-	FIIA

EBSL: extended-spectrum β-lactamase, CIM: Carbapenem Inactivation Method, ND: not deceted.
